# What Lies behind the Wish to Hasten Death? A Systematic Review and Meta-Ethnography from the Perspective of Patients

**DOI:** 10.1371/journal.pone.0037117

**Published:** 2012-05-14

**Authors:** Cristina Monforte-Royo, Christian Villavicencio-Chávez, Joaquin Tomás-Sábado, Vinita Mahtani-Chugani, Albert Balaguer

**Affiliations:** 1 Medicine and Health Sciences School, Universitat Internacional de Catalunya, Barcelona, Spain; 2 Institut Català d'Oncologia, Barcelona, Spain; 3 Gimbernat School of Nursing, Universitat Autònoma de Barcelona, Barcelona, Spain; 4 Research Unit, Hospital Nuestra Señora de Candelaria and Primary Health Care, Tenerife, Spain; 5 National Network for Biomedical Research in Epidemiology and Public Health, Instituto de Salud Carlos III, CIBERESP, Barcelona, Spain; 6 Centre de Recerca i Estudis Bioètics (CREB), Universitat Internacional de Catalunya, Barcelona, Spain; University College London, United Kingdom

## Abstract

**Background:**

There is a need for an in-depth approach to the meaning of the wish to hasten death (WTHD). This study aims to understand the experience of patients with serious or incurable illness who express such a wish.

**Methods and Findings:**

Systematic review and meta-ethnography of qualitative studies from the patient's perspective. Studies were identified through six databases (ISI, PubMed, PsycINFO, CINAHL, CUIDEN and the Cochrane Register of Controlled Trials), together with citation searches and consultation with experts. Finally, seven studies reporting the experiences of 155 patients were included. The seven-stage Noblit and Hare approach was applied, using reciprocal translation and line-of-argument synthesis. Six main themes emerged giving meaning to the WTHD: WTHD in response to physical/psychological/spiritual suffering, loss of self, fear of dying, the desire to live but not in this way, WTHD as a way of ending suffering, and WTHD as a kind of control over one's life (‘having an ace up one's sleeve just in case’). An explanatory model was developed which showed the WTHD to be a reactive phenomenon: a response to multidimensional suffering, rather than only one aspect of the despair that may accompany this suffering. According to this model the factors that lead to the emergence of WTHD are total suffering, loss of self and fear, which together produce an overwhelming emotional distress that generates the WTHD as a way out, i.e. to cease living in this way and to put an end to suffering while maintaining some control over the situation.

**Conclusions:**

The expression of the WTHD in these patients is a response to overwhelming emotional distress and has different meanings, which do not necessarily imply a genuine wish to hasten one's death. These meanings, which have a causal relationship to the phenomenon, should be taken into account when drawing up care plans.

## Introduction

For several decades now, clinicians and researchers have shown a growing interest in analysing the wish to hasten death (WTHD) in the context of serious or incurable illness. This phenomenon seems to affect a considerable number of patients, especially those facing the end of life or advanced stages of their illness [1], [2], [3]. In this regard, medical advances that increase life expectancy and disease chronicity, as well as other social phenomena found in developed societies (e.g. family or community breakdown), may contribute to making the WTHD more common [4], [5], [6], [7].

One of the difficulties faced by any clinical study of the WTHD is how to define the concept. Indeed, studies have not distinguished clearly between a general wish to die, the wish to hasten death and requests for euthanasia or physician-assisted suicide [8]. Thus, one finds the indistinct use of terms such as ‘wish to die’ [9], ‘want to die’ [10] or ‘desire to die’ [11], [12], as well as ‘wish to hasten death’ [13], [14], ‘desire for early death’ [15] and other related expressions or synonyms for requests for euthanasia or assisted suicide, such as ‘death-hastening request’ [16], ‘request to die’ [17], ‘request for euthanasia’ [18] and ‘request for physician-assisted suicide’ [19].

In addition to this lack of consensus regarding the conceptual definition and terminology of the WTHD, another aspect to consider is that the phenomenon tends to vary over time, depending on the stage or circumstances in which patients find themselves [11], [20], [21], [22], and this makes it enormously difficult to estimate its frequency. Nevertheless, some studies have sought to provide data regarding its epidemiology and prevalence in different settings [23], [24], [25]. Noteworthy in this regard has been the design of certain measurement instruments, such as the scale developed by Chochinov *et al.* [11], which was subsequently modified by Kelly *et al.* [26], or the instrument created by Rosenfeld *et al.* [27]. These scales should, in theory, facilitate the quantification and comparison of the WTHD in different populations, although the construct they quantify is often too broad and imprecise [20].

Another fundamental aspect that has been studied in relation to the WTHD is its aetiology. Factors addressed by research include pain [28], [29], depression [20], [30], [31], hopelessness [32], [33], [34], the feeling of being a burden [12], [34], [35] and loss of autonomy [28], [36], [37]. Various socio-cultural aspects may also play an important role in relation to the origin of such a wish, for example, family and social support or factors related to what gives meaning to life [38], [39], [40]. In general, clinical studies highlight a multi-factor aetiology, and the evolving literature on the WTHD points — perhaps in line with improvements in the treatment of physical pain — toward the considerable influence of other factors related with the spiritual and psychological dimensions of the individual [13], [14], [41], [42]. Whatever the case, any research of this kind faces the inherent challenge of how to access data in these contexts. Indeed, many of the data sources are indirect, for example, health professionals or the relatives and carers of the patient. Some studies do, however, report the attitudes or intentions of patients themselves in the early stages of their illness, presenting them with hypothetical scenarios of future suffering and asking them about their attitude towards such possibilities. In general, however, published quantitative studies select and evaluate different factors related to the WTHD, and in doing so limit and reduce the phenomenon to a small number of study variables [8].

Although quantitative research may provide highly valuable information about the WTHD it is difficult for such methods to fully penetrate the complex reality experienced by the patient who wishes to die [8]. Thus, there is a need for a more detailed approach to the meaning of the WTHD, one which helps to define its conceptual limits and to understand why such a wish may manifest. This is important because health professionals need to have knowledge and understanding of a patient's wish to die if they are to respond adequately to that person's needs. Such an understanding is also required when it comes to drawing up appropriate health and social policies. Given that the patient's perspective is key to providing this greater detail, qualitative research can make a significant contribution, as this method is specifically designed to understand subjective experience by focusing on the description and interpretation of the meaning of a given phenomenon, thereby enabling it to be explored in more depth [43], [44]. A number of studies have already used such a qualitative approach to analyse the wish to die from the viewpoint of the patient who expresses it [14], [45], [46].

The aim of the present study was to analyse, through an interpretative systematic review of qualitative studies, the meaning and motivation of the WTHD in patients with chronic illness or advanced disease [47], [48].

## Methods

### Design

**Figure 1:**
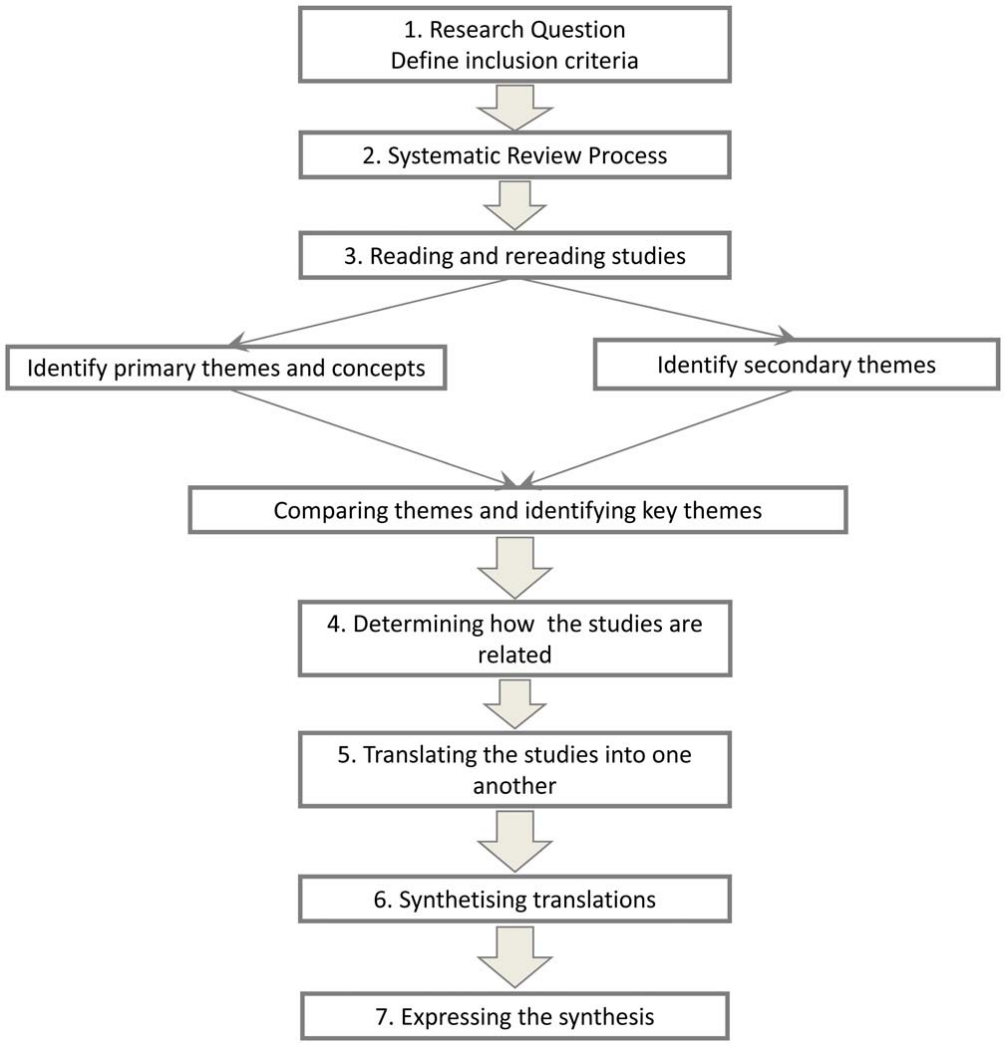
Meta-ethnography process according to Noblit and Hare.

Systematic review and interpretative synthesis, following the meta-ethnography approach developed by Noblit and Hare [49] (see Figure 1).

### Inclusion criteria

The criteria for sample selection required that the original studies described the ‘wish to hasten death’ in patients with a diagnosis of chronic or advanced disease, and that the data of these primary studies were gathered from the patient's own perspective. No language restrictions were placed on the search. In accordance with our protocol, which was designed a priori, the original reports had to have been conducted using a qualitative approach in relation to both data collection and data analysis [50]. However, despite the different methodological approaches or even philosophical underpinnings of studies in this field we followed other authors [51] in focusing on the substantive area addressed by the study rather than on the specific methodology used. Studies using mixed methods were eligible for inclusion provided it was possible to extract the findings derived from the qualitative research.

### Search strategy and study selection

Studies were identified primarily through protocol-based systematic searches of relevant electronic databases using terms and text words from Medical Subject Headings (MeSH). The MeSH terms used were ‘*suicide, assisted*’, ‘*euthanasia*’ and ‘*qualitative research*’. The text words used were *advanced disease*, *advanced cancer*, *advanced illness*, *chronic illness*, *chronic disease*, *desire to hasten death*, *wish to hasten death* and *end of life decisions*. These terms and text words were combined with one another. In order to minimize the likelihood of excluding important studies a certain degree of experimentation was required to develop an appropriate search strategy. Indeed, in an attempt to focus more precisely on qualitative studies a search was also undertaken using the Rochester qualitative filter that was adapted by Nesbit [52] from MEDLINE hedges developed by McKibbon and Walker-Dilks [53], and which has been used by other researchers [54], [55]. This filter was used in conjunction with terms relating to the topic of interest. Table 1 describes the final search strategy, which was adapted to the selected databases in accordance with the specific language used in each. Searches were conducted in Web of Science, PubMed, PsycINFO, CINAHL, CUIDEN and the Cochrane Register of Controlled Trials. The timeframe covered by the databases used in the search was from their inception to November 2009. The journals *Qualitative Health Research* and *Qualitative Research* were hand-searched for the period 1995–November 2009. References of included studies were also reviewed. This process was complemented by a search for key authors, i.e. searching specifically for primary qualitative studies carried out by leading researchers on the topic of WTHD.

**Table 1:** Final database search strategy.

1. Desire to hasten death/	30. Field studies/
2. Wish to hasten death/	31. Theoretical sample/
3. Euthanasia/	32. Discourse analysis/
4. Assisted Suicide/	33. Focus groups/
5. Decisions end of life/	34. Phenomenology/or ethnography/or ethnological research.mp. [mp = title, subject heading, abstract, instrumentation]
6. 1 or 2 or 3 or 4 or 5	35. (qualitative or phenomenol* or ethnon*).tw
7. Chronic disease/	36. (grounded adj (theor* or study or studies or research)).tw.
8. Chronic illness/	37. (constant adj (comparative or comparison)).tw.
9. Advanced disease/	38. (purpos* adj sampl*).tw.
10. Advanced illness/	39. (focus adj group*).tw.
11. Advanced cancer/	40. (emic or etic or hermeneutic* or heuristic or semiotics).tw.
12. 7 or 8 or 9 or 10 or 11	41. (data adj saturat*).tw.
13. 6 and 12	42. (participant adj observ*).tw.
14. Qualitative studies/or qualitative	43. (Heidegger* or colaizzi* or spiegelberg*).tw.
15. Interviews/or interview*	44. (van adj manen*).tw.
16. Case stud*	45. (van adj kaam*).tw.
17. Case studies/or case study	46. (merleau adj ponty*).tw.
18. 14 or 15 or 16 or 17	47. (Husserl* or giorgi*).tw.
19. 13 and 18	48. (field adj (study or studies or research)).tw.
20. Qualitative Studies/	49. (lived adj experience*).tw.
21. Phenomenological Research/	50. Narrative analysis.tw.
22. Ethnographic Research/	51. Discourse* analysis.tw.
23. Ethnonursing Research/	52. Human science.tw.
24. Grounded Theory/	53. Life experiences/
25. Exp* qualitative validity/	54. Convenience sample/
26. Purposive Sample/	55. Exp* cluster sample/
27. Exp* observational method/	56. Or/14–55
28. Content analysis/or thematic analysis/	57. 6 and 56
29. Constant comparative method/	58. 12 or 57

Key to abbreviations as used in Medline (PubMed):

* , truncation; tw, text word; adj, adjective.

**Figure 2:**
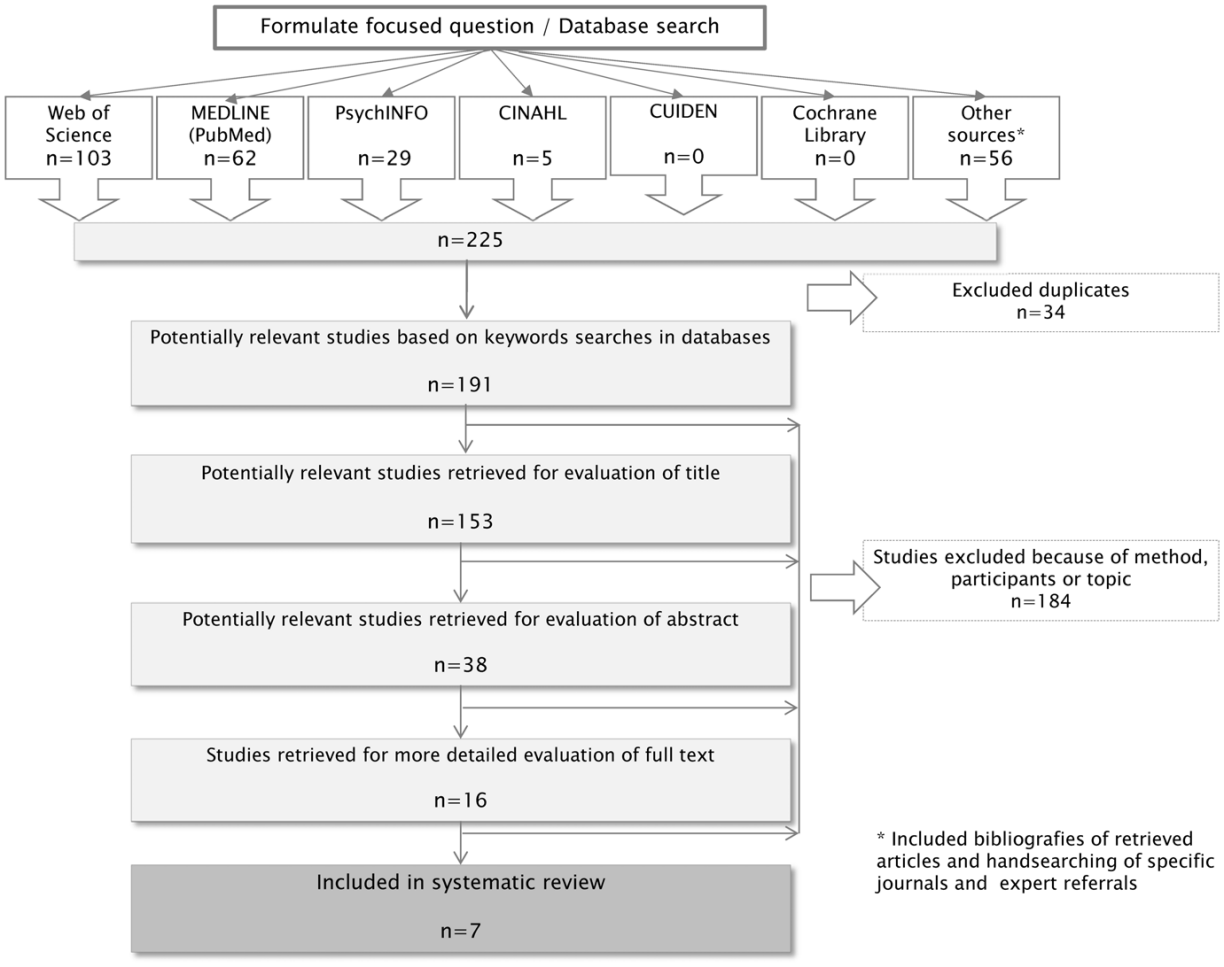
Flowchart of search results.

The lead researcher (CM) carried out the systematic literature search, which was then verified by another researcher (AB), who is an experienced systematic reviewer. The retrieved citations were sifted in three stages, as in a systematic review of quantitative studies. CM was responsible for reviewing the 191 citations retrieved, first by title, second by abstract and finally by full text. Studies were excluded when they did not meet the inclusion criteria. The results of this search were then fed back to another researcher (AB). Disagreements were resolved by discussion between the two reviewers and through reference to the full article. Finally, the research team agreed on the studies (n = 7) that should be included in the synthesis. Figure 2 illustrates the search process.

Studies were excluded if they were insufficiently focused on the topic and if the data were not gathered from the patients' perspective, although studies were included if they gathered data from the perspective of both patients and their family (only one of the selected studies). Studies were also excluded if they used qualitative data collection methods but not a qualitative method of analysis.

### Quality assessment

Although a wide range of quality assessment tools have been applied to qualitative studies [56], [57], [58], [59], [60], none of them has come to be regarded as a standard of reference [61], [62]. Here we decided to apply a Critical Appraisal Skills Programme (CASP) quality-assessment tool for qualitative studies [60], due to its extensive use among researchers [63], [64]. Two researchers (CM and AB) independently assessed the reporting of selected studies (Table 2). The full proforma document is available from the corresponding author.

**Table 2:** Methodological Quality of included studies assessed with CASP (1999): qualitative research checklist.

Reporting Criteria (CASP)	No (n = 7)	References of studies reporting each criterion
**1) Was there a clear statement of the aims of the research? ** ***Consider:*** (Yes//No//Comments)		
*– What the goal of the research was*	7	[13], [14], [45], [46], [69], [70], [71]
*– Why it is important*	7	[13], [14], [45], [46], [69], [70], [71]
*– Its relevance*	7	[13], [14], [45], [46], [69], [70], [71]
**2) Is a qualitative methodology appropriate? ** ***Consider:***		
*– If the research seeks to interpret or illuminate the actions and/or subjective experiences of research participants*	7	[13], [14], [45], [46], [69], [70], [71]
**3) Was the research design appropriate to address the aims of the research? ** ***Consider:***		
*– If the researcher has justified the research design (e.g. have they discussed how they decided which methods to use?)*	6	[14], [45], [46], [69], [70], [71]
**4) Was the recruitment strategy appropriate to the aims of the research? ** ***Consider:***		
*– If the researcher has explained how the participants were selected*	7	[13], [14], [45], [46], [69], [70], [71]
*– If they explained why the participants they selected were the most appropriate to provide access to the type of knowledge sought by the study*	7	[13], [14], [45], [46], [69], [70], [71]
*– If there are any discussions around recruitment (e.g. why some people chose not to take part)*	7	[13], [14], [45], [46], [69], [70], [71]
**5) Were the data collected in a way that addressed the research issue? ** ***Consider:***		
*– If the setting for data collection was justified*	7	[13], [14], [45], [46], [69], [70], [71]
*– If it is clear how data were collected (e.g. focus group, semi-structured interview etc)*	6	[14], [45], [46], [69], [70], [71]
*– If the researcher has justified the methods chosen*	3	[46], [69], [71]
*– If the researcher has made the methods explicit (e.g. for interview method, is there an indication of how interviews were conducted, did they used a topic guide?,)*	5	[14], [45], [46], [69], [71]
*– If methods were modified during the study. If so, has the researcher explained how and why?*	-	-
*– If the form of data is clear (e.g. tape recordings, video material, notes etc)*	7	[13], [14], [45], [46], [69], [70], [71]
*– If the researcher has discussed saturation of data.*	5	[13], [45], [69], [70], [71]
**6) Has the relationship between researcher and participants been adequately considered? ** ***Consider whether it is clear:***		
*If the researcher critically examined their own role, potential bias and influence during:*		
*– formulation of research questions*	3	[14], [45], [69]
*– data collection, including sample recruitment and choice of location*	5	[14], [45], [46], [69], [71]
*– How the researcher responded to events during the study and whether they considered the implications of any changes in the research design*	-	-
**7) Have ethical issues been taken into consideration? ** ***Consider:***		
*– If there are sufficient details of how the research was explained to participants for the reader to assess whether ethical standards were maintained*	6	[13], [14], [45], [46], [69], [70]
*– If the researcher has discussed issues raised by the study (e. g. issues around informed consent or confidentiality or how they have handled the effects of the study on the participants during and after the study)*	-	-
*– If approval has been sought from the ethics committee*	7	[13], [14], [45], [46], [69], [70], [71]
**8) Was the data analysis sufficiently rigorous? ** ***Consider:***		
*– If there is an in-depth description of the analysis process*	5	[14], [45], [46], [69], [71]
*– If thematic analysis is used. If so, is it clear how the categories/themes were derived from the data?*	6	[14], [45], [46], [69], [70], [71]
*– Whether the researcher explains how the data presented were selected from the original sample to demonstrate the analysis process*	3	[14], [45], [46]
*– If sufficient data are presented to support the findings*	7	[13], [14], [45], [46], [69], [70], [71]
*– To what extent contradictory data are taken into account*	-	-
*– Whether the researcher critically examined their own role, potential bias and influence during analysis and selection of data for presentation*	-	-
**9) Is there a clear statement of findings? ** ***Consider:***		
*– If the findings are explicit*	7	[13], [14], [45], [46], [69], [70], [71]
*– If there is adequate discussion of the evidence both for and against the researcher's arguments*	4	[14], [45], [69], [71]
*– If the researcher has discussed the credibility of their findings (e.g. triangulation, respondent validation, more than one analyst.)*	4	[14], [45], [69], [71]
*– If the findings are discussed in relation to the original research questions*	7	[13], [14], [45], [46], [69], [70], [71]
**10 How valuable is the research? ** ***Consider:***		
*– If the researcher discusses the contribution the study makes to existing knowledge or understanding (e.g. do they consider the findings in relation to current practice or policy, or relevant research-based literature?)*	7	[13], [14], [45], [46], [69], [70], [71]
*– If they identify new areas where research is necessary*	7	[13], [14], [45], [46], [69], [70], [71]
*– If the researchers have discussed whether or how the findings can be transferred to other populations or considered other ways the research may be used*	5	[14], [45], [69], [70], [71]

For a number of reasons no studies were excluded on the basis of quality. Some authors, such as Dixon-Woods et al. [65], claim that poor quality studies can nonetheless yield valuable insights, although other research suggests that such insights are not usually central to the overall understanding which can be sustained by good quality studies [66], [67]. Indeed, there is no consensus among researchers regarding the role of quality criteria and how they should be applied [65], [68]. The appraisals are included here as we considered that this may indirectly lead to improvements in the quality of reporting of qualitative research and meta-ethnography results. However, we did not aim to assess the quality of each study, as our intention was to assess the explicitness and comprehensiveness of reporting.

This research did not require the approval of our local ethics committee, since all the studies included in the review were already approved by their respective ethics committee.

### Synthesis

The seven qualitative studies were synthesized using Noblit and Hare's [49] seven-stage method, which makes systematic comparisons by translating studies into one another. In the first phase we identified the topic of interest that the qualitative studies might inform. The second phase involved selecting the studies for inclusion in the synthesis. Each paper was then read and re-read in order to draw up a list of key metaphors in each study and to identify common and disparate concepts and themes both within and across the studies. The purpose of this reading was to obtain a more in-depth knowledge of the papers and to recognize the different metaphors and concepts used in each. The findings sections of the research reports were divided into text units that were coded by words, sentences or paragraphs, according to content. These codes were then grouped into themes that defined characteristics or different dimensions of the phenomenon addressed by the study. Noblit and Hare's method [49] can be used to compare studies in a variety of ways, depending on the relationships between those studies. Here, and at the end of this phase, we decided that studies would be directly compared by means of *reciprocal translations*, that is, metaphors were written to express the similarities between study findings, with any exceptions being made clear. This method is appropriate when studies are essentially about similar issues [49]. The themes in each paper were initially identified by the main researcher (CM), and were later discussed and analysed by the whole research team. Disagreements were resolved by re-reading the full articles. Having identified the main concepts that emerged from each paper, a search was then undertaken for the presence or absence of these concepts in the seven papers. During this process the authors made sure that each key concept took on similar meanings in all the papers, although they also identified those which were unique or specific to one or more of the studies. The synthesis began with the earliest published paper, that of Lavery *et al.* [45], and then worked through the studies in chronological order of publication. The comparison process began with the themes identified in the first study, to which others were added as they emerged. At the same time, these themes were newly translated to the whole sample and to each individual study. The reciprocal translations enabled us to develop a table that shows each theme with its categories, as well as quotations from participants to explain each theme (see Table 3).

**Table 3:** Quotations from participants and authors of primary studies to illustrate each theme.

Themes/– Categories	Quotations from participants in primary studies	Interpretations of findings offered by authors
**Loss of Self:**		
– Loss of function	‘Um, the ability to perform simple things like you know, going to the bathroom on your own and not through a bag, um, breathing with your own lungs, not dependent upon a machine to keep the body parts functioning, um being able to do anything, I mean as long as you can think then you can live, but if you can't no longer even formulate a thought due to dementia or you know the ravages of the disease. You know, if you were to stand there in your former self, would you want to see yourself in that position? I know I wouldn't. You get to the point where there's no return, you know, I can understand somebody saying, well geez, you know, like I used to be somebody, but now, like I mean, you know, I'm no better than like a doll, somebody has to dress me and feed me and I guess it's uh, I don't know how to explain it, really’ [45].	Loss of self.
	‘There were many times when I was in such pain and such misery. I said, let me go… finished…no more of this torture’ [14].	The immediate situation was unendurable and required instant action.
	‘You don't know how much I am suffering. Come and deal with me; I need your attention and help’ [14].	
	‘You turn them over, they're in pain. They're going to shit themselves, they're going to piss themselves, they're going to lie there and have someone do all their bodily functions and just, they're going to suffer, the whole time, there's going to be no happiness, they're going to go down to 60–70 pounds, they're just going to, their whole last weeks of life is just going to be pain and agony and people coming in, people being upset, them being upset’ [45].	Disintegration.
	‘I'm inconveniencing, I'm still inconveniencing other people who look after me and stuff like that. I don't want to be like that. I wouldn't enjoy it, I wouldn't, I wouldn't. No, I'd rather die’ [45].	Disintegration. Symptoms and loss of function can give rise to dependency on others, a situation that was perceived as intolerable.
	‘I can't move, just lie here… feeling like a vegetable…a useless person… needing people to feed me’ [46].	Perception of suffering for self and significant to others.
	‘…the terrible weakness and the nausea and just not feeling like you can do anything. …And it's kind of like goals that I actually have or things that I want to accomplish are slowly being taken away… it's kind of like the realm of the possible…is shrinking’ [70].	Feeling weak, tired and uncomfortable. Illness-related experiences.
	‘There have been times I've felt so much a burden on my family that maybe it is best for me to die just to relieve them of going through the terminal phase of my disease’ [14].	A gesture of altruism.
	‘There were many times when I was in such pain and such misery. I said, let me go… finished…no more of this torture’ [14].	The immediate situation was unendurable and required instant action.
	‘You don't know how much I am suffering. Come and deal with me; I need your attention and help’ [14].	
– Loss of control	‘In the future when I can't manage, I would feel very bothersome and very suffering as if I'm really burdening them. I'm afraid of having others to serve me’ [46].	Anticipation of a future worse than death itself.
	‘…if I'm going to be rolling around in my own faeces because I have no control, then forget it’ [45].	Loss of self.
	‘Dignity is that I have control over my body, when, when, not not a virus that is going to take my life. I'm the one who's going to decide when my life will end, not a virus, and not with great pain. Not anything else other than in, in my control. It is my control, my choice to do’ [45].	
	‘When I'm in pain, it is not so much the pain, it's the loss of control and the helplessness’ [69].	Desire to hasten death as an expression of despair.
	‘I will do things my way and the hell with everything and everybody else. Nobody is going to talk me in or out of a darn thing…what will be, will be; but what will be, will be done my way. I will always be in control’ [68].	Desire for control.
	‘In the future when I can't manage, I would feel very bothersome and very suffering as if I'm really burdening them. I'm afraid of having others to serve me’ [46].	Anticipation of a future worse than death itself.
– Sense of ‘loss of dignity’	‘I think we should all be allowed to die with our dignity intact’ [45].	Participants' experience of disintegration.
	‘No matter how much they love you, you are always a burden. You automatically become a burden to everyone. Even to your own missus’ [13].	Being perceived as a burden to others.
	‘…not wanting to be seen by those that love me as this skin-and-bone frail, demented person. In other words, I don't want that image of me for me, and I don't want that image to be kind of a last image that my daughters and loved ones have of me. And that's just a dignity issue’ [70].	Loss of sense of self.
	‘I'm not comfortable, and I can't do anything, so as far as I'm concerned in quality of life I'm not living; I'm existing as a dependent non-person. I've lost, in effect, my essence’ [70].	
	‘Oh, it's the dignity and wholeness of my body, as well as spirit. And, it is, it's cruel too for others to have to do this when there's no end in sight, other than death. To just, to clean me up. I just don't want that…’ [45].	Loss of self.
	‘After a while, your family, who you love so dearly, will remember you as a washed-out role model… It will remind them of what they have to go through, the lack of strength, the weakness, and so forth’ [14].	The dying process itself was so difficult that an early death was preferred.
	‘You've become a bag of potatoes to be moved from the spot to spot, to be rushed back and forth from the hospital, to be carried to your doctors' appointments or wheeled in a wheelchair, and it really does take away any self-worth, any dignity, or any will to continue to live’ [45].	Disintegration.
	‘I think we should all be allowed to die with our dignity intact’ [45].	Participants' experience of disintegration.
– Loss of meaning	‘There's not any good reason for me to go on living. Nobody really needs me… I'm really not serving any purpose. If you don't, aren't needed by anybody, you kind of have a different feeling about life’ [71].	Psychosocial factors (useless, boredom, burden, lack of enjoyment in life) motivating the serious consideration of a hastened death.
	‘I'm just saying to myself when I go to sleep, ‘Just let me die.’ I don't want to have to wake up and face this… honestly I just pray that I would just die in my sleep. I have nothing to live for, absolutely nothing. There's nothing coming up in my life that I am living towards, and if there was it would be so terrible because it probably wouldn't happen’ [69].	Desire to hasten death as an expression of despair.
	‘One daughter explains about her mother: “The things that were meaningful to [my mother] in her life were her art, her ability to do her art and her friends, and spending time with her friends and cooking and eating. And she was…very convinced that when she couldn't do any of those things anymore, her life would be meaningless, and she wouldn't want to live anymore’ [70].	Loss of function.
	‘There's not any good reason for me to go on living. Nobody really needs me… I'm really not serving any purpose. If you don't, aren't needed by anybody, you kind of have a different feeling about life’ [71].	Psychosocial factors (useless, boredom, burden, lack of enjoyment in life) motivating the serious consideration of a hastened death.
	‘I'm just saying to myself when I go to sleep, ‘Just let me die.’ I don't want to have to wake up and face this… honestly I just pray that I would just die in my sleep. I have nothing to live for, absolutely nothing. There's nothing coming up in my life that I am living towards, and if there was it would be so terrible because it probably wouldn't happen’ [69].	Desire to hasten death as an expression of despair.
**Fear:**		
– Fear of dying process	‘It'll be extremely terrible. It'll be coming up from there, just everywhere. I mean the complications and that would give me so much pain and suffering. I anticipate the future would be like this. Very severe, very scary when I think about it’ [46].	Anticipation of a future worse than death itself.
	‘I, I fear some of the, uh, some of the physical stress that may come in the course of my dying. Nobody chooses to die little by little. At least, I can't visualize that’ [71].	Factors motivating the serious consideration of a hastened death.
	‘I can't bear the dying process so I'll short circuit it by dying’ [14].	The dying process itself was so difficult that an early death was preferred.
	‘I don't want to go through the dying process so I'll kill myself’ [14].	
	‘It'll be extremely terrible. It'll be coming up from there, just everywhere. I mean the complications and that would give me so much pain and suffering. I anticipate the future would be like this. Very severe, very scary when I think about it’ [46].	Anticipation of a future worse than death itself.
– Fear of imminent death	‘Not much hope, nor would there be any miracles…You doctors can't help when the patients deteriorate and then drop dead…’ [46].	Reality of disease progression.
	‘This sort of disease ultimately leads to death. I have to walk that path’ [46]	
	‘I haven't been in hospital before. I wouldn't know the facts. I haven't been ill before’ [46].	Anticipation of a future worse than death itself.
	‘…the end of many dreams for, plans, complete halt to things I was doing, want to do. The biggest thing is the weakness, which I absolutely hate, not being able to do things, to realise that this is virtually the end of it all. There's no future really. You can't plan anything’ [13].	Impact on the patient.
	‘Not much hope, nor would there be any miracles…You doctors can't help when the patients deteriorate and then drop dead…’ [46].	Reality of disease progression.
**WTHD as a desire to live but not in this way; WTHD as a sort of cry for help:**		
	‘The goal is now to die. …I'm using my flexibility not to devote my time toward how I am going to die and praying, etc… I'm using my flexibility in time management to do things that the living do, not the dying’ [14].	A manifestation of the will to live.
	‘Wish to live but can't live; wish to die but can't die’ [46].	Perception of suffering for self.
	‘See, there's a problem while planning or pursuing your death… On the one hand, I am saying all these things, and, on the other hand, I am going down for radiation¡ [14].	A manifestation of the will to live.
	‘I've experienced such incredible pain over the last little while and more in the last week. Such incredible pain that it made me think that death is preferable to this…I'll sit there for 2 hours in terrible pain. Such pain where I can't yawn even, and I get only half a yawn and my whole insides turn and waiting for the medication to start to work… I'd love to have 48 h let's say, I'd love to have this weekend where I could plan to have a nice weekend and have no pain. I'd love to do that and it doesn't happen, and the pain affects everything. It makes you tired. It affects how you can eat. It affects your mood. It affects other people, and the fact is that even if you try to hide it, you can't… So that's hard…and I know it's gonna get worse, so that's hard too. It's great to be alive, and pain takes that life out of you, and to sit there for 2 hours with a blanket around your just shivering, with no solution, is really hard’ [69].	Desire to hasten death as an expression of despair.
**WTHD as a way for ending suffering:**		
	‘I can't bear the dying process so I'll short circuit it by dying’ [14].	The dying process itself was so difficult that an early death was preferred.
	‘I feel, deep inside, I don't want to feel hurting… that I want to end this… I ask God why he don't take me, why I suffer so much’ [14].	The immediate situation was unendurable and required instant action.
	‘In a sense it's artificial that I'm still alive. Even a few years ago that would not have been the case for me to survive that long, but there are limits to what any organism will take or can do, and I have reached my limit’ [69].	Desire to hasten death as a manifestation of letting go.
	‘Pain is my biggest fear. It puts me in a darkness and a lack of will to go forward and a desire to die… The pain wants me to have vehicle to just, just stop my life’ [14].	A hastened death was an option to extract oneself from an unendurable situation.
**WTHD a kind of control, ‘to have an ace up one's sleeve just in case’:**		
	‘If I had to go through [an episode of acute shortness of breath] again, I would throw myself in front of a subway train. I am not going through that again’ [14].	A manifestation of the last control the dying person can exert.
	‘If the pain gets worse, then I want to be dead’ [14].	A hastened death was an option to extract oneself from an unendurable situation.
	‘I just feel sometimes as though cancer is, uh, an opponent. And, it seems to me, it says to itself, ‘I am in control of this body. This is mine, I will do whatever I want to with it’” [71].	Sense of control; ultimate control through physician-assisted death.

The reciprocal translations were then brought together by synthesizing them, starting from the identified themes and matching them with their respective quotations. This process involved further re-readings of the original studies, with the final themes obtained being once again compared at the end of the reciprocal translation process. This gave rise to what Noblit and Hare [49] refer to as the ‘line of argument’. In this phase it was possible to re-conceptualize the findings, generating a new interpretation of the phenomenon explained by the data and leading to a synthesis that not only represents more than the sum of its parts but also preserves the integrity of each of the individual studies. This process was initially carried out by the first author, although conceptualizations of the emergent themes were newly discussed and considered in research team meetings, in which any disagreements were resolved by considering the background of the different research. Finally, an explanatory model of WTHD in patients with advanced disease was obtained.

## Results

### Description of included studies

The main features of the seven studies included in this synthesis are summarized in Table 4. These studies were published between 2001 and 2009, and were conducted in Canada [45], [69], the USA [14], [70], [71], Australia [13] and China [46]. They all explored the wish to hasten death from the patient's perspective and were conducted within a clinical or health service setting. Although all the studies justified their use of the qualitative approach, only four of them [14], [45], [46], [69] specified the underlying theoretical framework; these four papers also specified the role of the researcher. The method of analysis was described in five papers [14], [45], [46], [69], [71]. Interview questions and prompts were not provided in all the articles. Studies generally provided insight into the experiences, perceptions and views of patients.

**Table 4:** Characteristics of included studies in the review.

Source paper	Country setting	Participants	Research Design	Data collection	Setting	Sampling	Data collection
Lavery *et al.* 2001 [45]	Ontario, Canada	31 men and 1 woman with HIV or AIDS	Grounded Theory	In depth interviews	HIV Ontario Observational Database (HOOD), which is a provincial epidemiological database.	Purposive sampling.	October 1996 to september 1997
Kelly *et al.* 2002 [13]	Brisbane, Australia	30 terminally ill cancer patients, who endorsed some wish to hasten death	Mixed-Methods Study. Qualitative method: Descriptive qualitative study	Quantitative scale and Semi-structured interviews	Inpatient hospice unit and home palliative care service	Purposive sampling	Between 1998 and 2000
Coyle & Sculco 2004 [14]	New York, USA	7 terminally ill cancer patients that expressed to desire to hasten death	Phenomenology	In depth interviews	Pain and palliative care unit in an urban center cancer research	Purposive sampling	1–6 interviews to each patient in 6 months
Mak & Elwyn 2005 [46]	Hong Kong, China	6 patients that requested to hasten death	Hermeneutic Phenomenology	In depth interviews	Palliative care unit consisted of a 26-bedded hospice in China. This unit followed the UK model of palliative care with a multi-disciplinary team	Purposive sampling: theoretical	4 months period in 2000
Pearlman *et al.* 2005 [70]	Seattle, USA	35 patients who seriously pursued a hastened death	Descriptive qualitative study	Semi-structured interviews	Patient advocacy organizations that counsel persons interested in PAS, hospices and grief counselors	Purposive sampling	April 1997 to march 2001
Schroepfer 2006 [71]	Wisconsin, USA	18 terminally ill elders (50 or more years old) who desire to hasten death	Content analysis; inductive method in locating themes and patterns	Face-to-face interviews	2 palliative care programs, 2 hospital outpatient clinics and 6 hospices	Purposive sample	Not specify
Nissim *et al.* 2009 [69]	Toronto, Canada	27 ambulatory patients with advanced lung or gastrointestinal cancer	Grounded Theory	Semi-structured interviews and discovery- oriented	Outpatient clinics at a large cancer center in Toronto	Theoretical Sampling	March 2003 to November 2006

### Description of themes

Six major themes emerged when synthesizing the translations: the WTHD in response to physical/psychological/spiritual suffering, loss of self, fear, the WTHD as a desire to live but ‘not in this way’, the WTHD as a way of ending suffering, and the WTHD as a kind of control over life, ‘to have an ace up one's sleeve just in case’. Overall, the WTHD emerges as a phenomenon that does not necessarily imply the wish to die, and it appears as a response to an overwhelming emotional distress among patients in the advanced stages of disease. Table 5 illustrates the themes identified in each study.

**Table 5:** Themes identified in each study.

Themes/– Categories	Study Reference						
	[13]	[14]	[45]	[46]	[69]	[70]	[71]
**Wish to hasten death in response to physical-psychological-spiritual Suffering**	Yes	Yes	Yes	Yes	Yes	Yes	Yes
**Loss of Self:**							
– Loss of Function	Yes	Yes	Yes	Yes	Yes	Yes	Yes
– Loss of Control	Yes	Yes	Yes	Yes	Yes	Yes	Yes
– Sense of “Loss of dignity”	--	Yes	Yes	Yes	Yes	Yes	--
– Loss of Meaning	Yes	Yes	--	--	Yes	Yes	Yes
**Fear:**							
– Fear to dying process	Yes	Yes	Yes	Yes	Yes	Yes	Yes
– Fear to imminent death	Yes	Yes	--	Yes	--	--	Yes
**WTHD: as a desire for living but “not in this way”; as a sort of “cry for helping”**	--	Yes	Yes	Yes	Yes	Yes	Yes
**WTHD: as a way for ending suffering**	Yes	Yes	Yes	Yes	Yes	Yes	Yes
**WTHD: a kind of control of my life “To have an ace up one's sleeve just in case”**	--	Yes	Yes	--	Yes	Yes	Yes

### Desire to hasten death in response to physical/psychological/spiritual suffering

In all the selected studies the WTHD is explained as a complex phenomenon, of multi-factor aetiology, that is almost always triggered by the exacerbation of physical and/or psychological symptoms, leading to a situation of emotional distress and hopelessness. All the patients featured in the seven studies presented physical, psychological and spiritual suffering, and the WTHD emerged as a response to this.

### Loss of self

The theme *Loss of self* reflects a response to *loss of function*, *loss of control* and *loss of meaning*.

The loss of body function was a common denominator among all participants and contexts (HIV patients, cancer patients, palliative care in-patients and elderly patients). As the disease evolves it brings physical deterioration, weakness and various physical symptoms, and this is accompanied by a progressive loss of body function, whether in terms of an inability to go to the toilet, urinary or faecal incontinence, or difficulties eating or even breathing. The deterioration in body function was described by all the participants in the various studies as a negative experience, mainly in terms of the physical dependency that it implied. This dependency in relation to the simplest of tasks appears to be related to hopelessness and emotional distress. The perception of loss was intrinsically linked to the physical changes that were produced in all the participants of the seven studies. Incontinence and/or being dependent on others for help in using the toilet was described as an especially significant event that preceded or accompanied the WTHD.

The loss of body function leads, in turn, to a loss of the different roles that the person had acquired in life (professional, social, family, etc.), these being replaced by the new role of ‘dependent individual’. Indeed, functional deterioration and dependency restrict not only the person's professional capacities but also the possibility of maintaining his/her social relationships or role within the family; for example, a person may cease to be the mother or father who looked after the family and become a patient who needs to be cared for.

Alongside the loss of body function that occurs as the disease progresses there is also a loss of control. In the studies reviewed this loss of control was interpreted in two ways. On the one hand there is loss of control over the body, which is linked to a decline in various physical functions (difficulty getting up unaided, with walking or eating, with sphincter control, etc.). However, this is accompanied by another, more internal loss of control, namely the control over one's own life and future. The loss of autonomy caused by dependency invariably leads the individual to feel that he/she is a burden to the family or caregivers, and prior to or alongside the emergence of this feeling the majority of patients say they feel useless. This sense of uselessness and of being a burden was occasionally expressed in terms of a ‘sense of loss of dignity’ [14], [45], [46], [69], [70], [71], although the term ‘dignity’ was understood by the participants as *not being able to do anything unaided*, and is therefore closer to a sense of dependency. Similarly, the patients sometimes alluded to the idea that life as they were living it was ‘not dignified’, and they went on to say that they *weren't like that before*, and that *they didn't want people to remember them in this way*, i.e. as fragile and dependent. This perception led some patients to feel a ‘*loss of meaning*’ [14], [46], [69], [70], [71]. All these aspects lead to a progressive undermining of how patients see and regard themselves, and this would constitute a *Loss of self* [14], [46], [69], [70], [71].

The study by Lavery *et al.* [45], conducted with AIDS patients, is the only one to have described a meaning for the *Loss of self* that is not covered by the above definitions. In addition to a loss of body function the participants in this study also expressed a loss of community, self-exclusion, a fear of social rejection and existential isolation. Lavery *et al.* [45] linked this loss of community to society's rejection of AIDS sufferers and suggested that its emergence has much to do with the lack of social support given to these patients.

### Fear

The theme *Fear* emerges in the interviews with the majority of participants in the studies reviewed, and it was identified with two related categories: *Fear of the dying process* and *Fear of imminent death*.

Upon becoming aware of their prognosis the participants came to regard their own physical deterioration as being worse than death itself, this leading to a *Fear of the dying process*. The reasons for this fear fall into two groups: physical and psycho-social. Physical reasons include the fear of pain, the exacerbation of signs and symptoms, and a progressive deterioration in functional capacity, coupled with the fear that all this will become unbearable. Their previous experiences of pain and suffering led all the patients to fear that such experiences would be repeated. The suffering of relatives and/or ill acquaintances also led patients in three of the studies [46], [70], [71] to be fearful of the future. As for psycho-social reasons these took the form of foreseeing the loss of role, greater dependency and the fear of being a burden. These thoughts about a future with greater suffering were described by participants as being overwhelming and caused them enormous anguish.

The theme *Fear of imminent death* emerges when patients become aware of the proximity of their own death. The acknowledgement of death's inevitability, coupled with the awareness that there was no way back from their situation [13], [14], [69], produced much anguish among participants and in many cases led to a feeling of hopelessness [14], [46], of having no options left for the future [13], and to a sense of *being in a dark tunnel, without seeing any light* or a feeling of *paralysis* [69].

### WTHD as a way of ending suffering

Among participants in the studies included, the WTHD also emerged as a way out, and in some cases [45], [69] as the only way of ending their physical and psychological suffering. Death was not considered as an aim in itself, but rather as an escape. Indeed, the idea of putting an end to their life brought a sense of relief to some patients.

In the study by Schroepfer [71] the WTHD was regarded as a way out or as a means of relieving loneliness, fear, dependence, a lack of hope and the feeling that life was no longer enjoyable. The study by Nissim *et al.* [69] suggested that in the face of oppression and despair, death could be seen as the only alternative, with the WTHD being the essence of a plan to relieve suffering. Similarly, Lavery *et al.* [45] reported that the WTHD was seen by participants as a means of limiting disintegration and loss of self.

In five of the studies reviewed [14], [46], [69], [70], [71] the participants also described the WTHD as a way of reducing the suffering being caused to family and carers. Coyle and Sculco [14] interpret this as *a gesture of altruism*, since the WTHD is motivated by a desire to relieve the family of the burden of care and of witnessing their relative's progressive deterioration. However, although the WTHD was driven by such a motive in some patients [71], in others (or simultaneously in the former patients) the desire to cause no more pain to their relatives led them to precisely the opposite conclusion, i.e. they repressed the WTHD. As such, their wish to protect their family took precedence over their own wish to hasten death [71].

### WTHD as a desire to live but not in this way; WTHD as a sort of ‘cry for help’

Rather than manifesting as a genuine wish to die the WTHD appears more as a desire to live but not in this way. With only two exceptions all the participants in the studies included showed through their behaviour a desire to live. For example, they agreed to continue with medical treatment until the end of their life, and many of them said they wanted to be treated as *a living person, not as someone on their deathbed* [14], [46], [71]. The only two behaviours which seemed to contradict this were those of participants 3 and 7 in the study by Coyle *et al.* [14] (participant 3 accepted transfer to a terminal care hospital, stating that this would hasten his death because he would no longer be in a kind of life-prolonging care, while participant 7 refused surgery that could have prolonged her life for several years without significant morbidity). The authors also interpreted these behaviours as contradicting the rest of their findings.

The apparent paradox between the desire to live and the WTHD is highlighted by Coyle and Sculco [14], whose findings clearly illustrate the will to live among their participants. Similarly, Mak and Elwyn [46] also note that despite expressing the WTHD, their participants hoped to receive holistic and good quality end-of-life care. An example is that of patient 4, who said: “*You want to be cured, that isn't possible, but at least give me back some of my energy*” [46]. It is also noteworthy that those patients in the study by Schroepfer [71] who expressed the WTHD also showed through their behaviour a desire to go on living; indeed, they themselves said that if they had not yet acted on the wish to hasten their death this was because, despite their deterioration, there was still something that gave meaning to their life.

The WTHD also emerges a cry for help in the face of suffering. The participants were aware of the precarious and extreme nature of their situation, which was experienced as unbearable and as something that required immediate action [14], [45], [46], [69]. The patients' WTHD manifests in the different contexts as a way of letting others know what they are going through, and at the same time as a call for help in bearing the situation. Behind each WTHD one finds hidden desires for understanding and for someone to accompany them in their suffering and in the process of mourning for what has already been lost [14], [46], [69], [71].

### WTHD as a kind of control, ‘to have an ace up one's sleeve just in case’

Most participants considered the possibility of their own death as a kind of control over their life [14], [45], [69], [70], [71]. The wish to determine one's own death would be an extreme (or perhaps the sole) manifestation of the desire for control. When the participants perceived that they had very little left, and that they no longer had any control over many aspects of their life, the potential to decide how and when to die was seen by some of them as being all that remained of their autonomy, as if it were the last card left to play. In general, those patients who had decided to hasten their death, as a way of reaffirming their ability to make their own decisions, reported feeling more able to tolerate the pain of the present and the uncertainty of the future [14], [45], [69], [71].

The desire for control was also expressed as a kind of safety net, or what Coyle and Sculco [14] called an *if-then* situation. Several of the participants turned to control strategies such as storing up barbiturates, fantasizing about planning their own death through a trip to the Netherlands (where it would be easier to achieve), or imagining that they would throw themselves into the sea despite not knowing how to swim. For them, the sense of control comes from having a hypothetical exit plan [14], [69], [71], akin to having an ace up their sleeve just in case, because at the end of the day they did not act on it.

A slightly different aspect of this longing for control is when the WTHD appears as a way of manipulating the patient's surroundings. In this regard, Coyle and Sculco [14] interpret some patients' explicit declaration of the WTHD as being an attempt at manipulation of the family to avoid abandonment. In this case, the WTHD would follow from what is probably an unconscious desire to control the surroundings, with the patients finding no other way of doing so.

### Explanatory model

**Figure 3:**
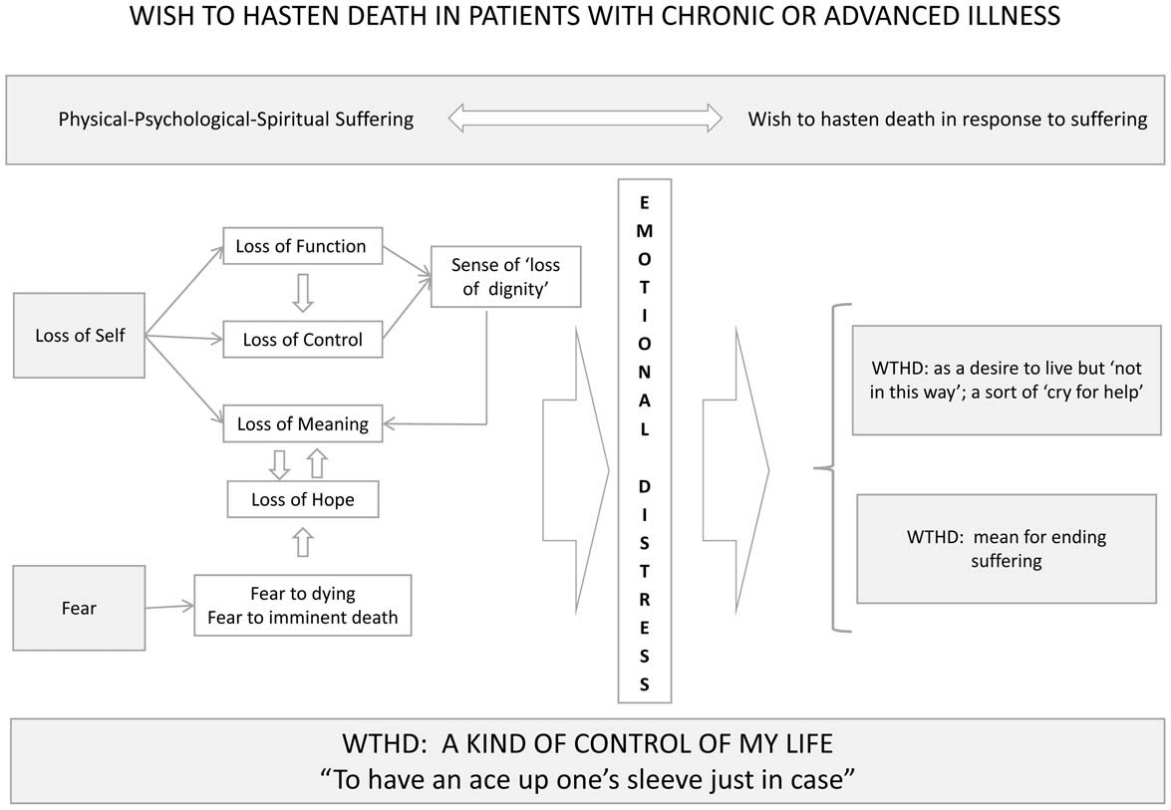
Line of argument: Explanatory model of WTHD.

The explanatory model derived from these results showed the WTHD to be a reactive phenomenon, a response to multidimensional suffering, rather than simply being one aspect of the despair that may accompany this suffering. According to this model the factors that lead to the emergence of a wish to hasten death are total suffering, loss of self, and fear, which together produce an overwhelming emotional distress in relation to which the WTHD is seen as a way out, i.e. the individual wishes to cease living in this way and to put an end to suffering while maintaining some control over the situation. As such, the model suggests a new meaning for the WTHD and highlights the factors related to — or which are the cause of — this wish. Although this conceptual model was developed on the basis of reports by hospice patients, those with cancer or HIV and elderly patients the fact that it was obtained by re-interpreting meanings across a number of different qualitative studies means that it may also be applicable to the experiences of other people living with chronic illness. The findings of the whole synthesis are summarized in Figure 3.

## Discussion

This synthesis suggests that the wish to hasten death (WTHD) is a multifactorial construct with multiple meanings that do not necessarily imply a genuine desire to hasten one's death or actually taking steps towards this. Rather, it is a phenomenon that appears, among patients in the advanced stages of illness, as a response to the extreme suffering that affects all aspects of their human existence.

The first stage of the analysis, *reciprocal translation*, revealed the themes that explain the meaning of the WTHD in these patients. The subsequent *line of argument approach* enabled us to develop an explanatory model which shows the WTHD to be a reaction to overwhelming emotional distress, rather than simply one aspect of the despair that may accompany this suffering.

Of the six main emergent themes, the first, i.e. the WTHD in response to physical/psychological/spiritual suffering, defines the WTHD as a response to this kind of suffering. The second theme, loss of self, is related to the gradual loss of physical, psychological and spiritual capabilities which the patient experiences. Alongside the third theme, i.e. fear of the dying process, this loss of self can generate a sense of hopelessness and emotional distress. Another of the defining characteristics of the loss of self in the present synthesis was a perceived ‘loss of dignity’, associated with the loss of autonomy and control. The same term was also used by Chochinov *et al.* [72], who developed the Dignity Model [73] and noted that the concept of dignity is not always used in the same way in the literature. The notion of dignity is usually regarded as an internal moral quality that is an inherent part of human life [74], but when the notion is seen in terms of individual autonomy then the patient's increasing dependence as the end approaches is experienced as a loss of dignity that undermines the value ascribed to life [75]. At all events the elements that comprise the model of Chochinov *et al.* (who found that those patients whose sense of dignity was compromised reported a stronger desire for death [73]) would refer to constitutive characteristics of the loss of self, similar to those found in the present study. Thus, entering a state of severe dependence would be interpreted by patients and those around them as something ‘undignified’, despite the fact that a degree of dependence is an inherent part of the human condition [76].

The meaning of life, another element of the theme ‘loss of self’, has recently attracted considerable interest among clinicians and researchers in the field of palliative care, and has become a key element of certain psychotherapeutic interventions [33], [39], [77]. Those patients who, despite their illness, continue to feel that life is meaningful are able to regard their life as worth living [78]. By contrast, a lack of meaning in life has been described as a factor associated with hopelessness and the WTHD, or even a request for active euthanasia [20], [79], [80], [81], [82].

The fourth theme identified in this synthesis, i.e. the WTHD as a way of ending suffering, emerges when the patient feels that almost everything has been lost, and that all that remains is suffering. Faced with such a situation, death appears to be the only way out for these patients.

The fifth theme, the WTHD as a desire to live but not in this way, expresses a veritable paradox, a sort of cry for help, since at the same time as expressing their wish to die these patients are seeking help and companionship. This is consistent with the findings of Dierckx de Casterlé *et al.* [83], who analysed the experiences of nurses in relation to patients who requested euthanasia. The study found that once they felt listened to and accompanied many patients ceased to request their own death.

The final theme, the WTHD as a kind of control over one's life, emerged in all the patients studied and implied, at times, a degree of planning one's own death; however, this was rarely transformed into action, and was more akin to ‘having an ace up one's sleeve just in case’. The desire for control, expressed here as the WTHD, would manifest the need to retain a degree of autonomy and capacity for decision making. However, although this control has been regarded as an active or purposeful element it would, in this case, refer more to a control over death, not over life. Other studies have also noted the importance of control in similar contexts, including cancer patients, those with neurodegenerative disease [9], nursing home patients [76] and those in palliative care, all of them expressing the need for control by taking an active role in decisions regarding their own care. This suggests that contemplating the need for an individual to have some control over the central aspects of his or her care should always form part of the design and implementation of any comprehensive care plan for these patients.

There was one concept which emerged during the synthesis that was described only in the study by Nissim *et al.* [69], and which the method of Noblit and Hare [49] clearly identified as being unique and specific. The study by Nissim *et al.* offered another point of view regarding the WTHD, which once again was not a genuine wish to hasten death but, rather, a manifestation of what the authors called *letting go*. This situation emerged only in the last weeks of life, during the final stage of illness in some patients, in whom their deterioration and tiredness led them to capitulate and give up. One might regard this as *throwing in the towel*, in other words, these patients accept their imminent demise once they acknowledge that death can no longer be resisted; at all events they were too tired to go on fighting. As the authors note [69], this situation could be compatible with the final stage of accepting one's illness, or in this case death, as in the model of Kübler-Ross [84].

The present synthesis also yielded an explanatory model of the WTHD. Although there are certain differences, other authors have previously described similar models (or syndromes) in an attempt to explain the hopelessness and distress felt by patients nearing the end of their life. For example, Dame Cicely Saunders [85], the founder of the modern hospice movement, coined the term *total pain* to refer to the suffering caused not only by physical pain but also by its psychological, social and spiritual counterparts, relating it to the numerous losses experienced by her patients. Similarly, Clarke and Kissane [86] have described what they call ‘demoralization syndrome’ as being *a clearly defined syndrome of existential distress occurring in patients suffering from mental or physical illness, specifically ones that threaten life or integrity of being*. They suggest that the syndrome is triggered by a feeling of being unable to cope with a stressful situation, and leads to hopelessness, helplessness, incompetence, isolation, a sense of failure, disheartenment, low self-esteem, a sense of meaninglessness and existential distress [80], [86]. Hence, demoralized patients may wish to die, but not in the same way as someone who has led a full life may then await its end; rather, the demoralized patient would be eager to die in the context of the anguish felt about a life not worth living. In these situations the wish to die would emerge as yet another manifestation of demoralization syndrome. Similar findings have been reported in another study that analysed the wish to die in a sample of patients with amyotrophic lateral sclerosis [9]. Those individuals who expressed the wish to die were more likely to meet criteria for depressive disorders and showed less optimism, found less comfort in religion, experienced greater hopelessness and suffering, and reported a loss of interest in living, the absence of pleasure and a loss of interest in activity. The authors attributed this wide range of symptoms to what Rosenfeld *et al.* [82] called the *syndrome of end-of-life despair*. The wish to die would therefore be one aspect of this end-of-life despair, analogous to the way in which Clarke and Kissane [86] related it to demoralization syndrome.

These syndromes, i.e. *total pain* [85], *demoralization syndrome* [86] and *end-of-life despair* [82], would appear to refer to a similar reality and they all seem to evoke a similar response in the patient: emotional distress. However, although each of their descriptions includes and explains the WTHD as an element of the syndrome the model that emerges from the present synthesis suggests that the WTHD should be regarded as a reactive phenomenon, a response to multidimensional suffering, rather than just another manifestation of a syndrome.

### Study limitations

Given the known correlation between depression and the WTHD [20] the fact that depression was not a variable explored in all the primary studies constitutes a limitation of the present synthesis. Three of the studies included in this review [45], [46], [71] make no reference to this aspect at any point. Coyle *et al.* [14] do acknowledge that depression is one of the factors that may lead to the WTHD, but they do not then analyse this variable in their sample of patients. The patients in the study by Pearlman *et al.* [70] presented neither depression nor hopelessness, although the authors recognize that their findings are limited by the nature of their study population, i.e. volunteers in advocacy organizations providing support to people who wished to die. The study by Kelly *et al.* [13] does not rule out the presence of depression among its participants, with the authors suggesting that they could have presented a depressive mood state given the low levels of life satisfaction they reported. Nissim *et al.* [69] report the presence of hopelessness in all their patients and, like Kelly *et al.* [13], consider the possibility that the signs and symptoms shown could be compatible with the demoralization syndrome described by Kissane *et al.* [80].

As regards our search strategy, the fact that the MeSH term ‘Qualitative Research’ was only introduced in 2003 could have limited the exhaustiveness of the search. However, the search strategies used were highly sensitive, including hand searches of reference lists, and we therefore believe that the review has covered all the relevant literature. Another possible limitation of the study is methodological in nature and relates to the fact that the studies included make use of different qualitative designs. In this review, however, the focus was on the substantive area addressed by each study. At all events, various authors [48], [87] have argued that this does not necessarily constitute a limitation, and in fact it could bring richness to the interpretative synthesis.

Further research is needed to determine the extent to which the present findings, which refer to the experience of the WTHD in terminally ill cancer patients, elderly patients, and AIDS and palliative care in-patients who were receiving care in a large urban cancer or AIDS centre, could be generalizable to other chronically ill individuals. It must also be acknowledged in this regard that all the studies included were conducted in developed — and mostly Western — countries, and even the study by Mak and Elwyn [46], which was carried out in China, was based on the Western model of hospice care. The literature search retrieved no studies on the WTHD that had been conducted in Africa, Central or South America, or even in European contexts, where family and other social factors could play different roles [40]. It should also be noted that the sample characteristics, in terms of age, sex, prognosis and socio-economic status, etc., were not always formally described in the studies included, thereby hindering the transferability of results.

### Implications for policy and practice

The results highlight the importance of analysing the meaning which patients in the advanced stages of an illness attribute to their suffering and its consequences, which render them highly vulnerable. Any manifestation of the WTHD should be carefully assessed, with the possible reasons for it being considered in relation to the six themes described here. The results also suggest the need to develop comprehensive care plans that facilitate communication and enable carers to explore the possibility of an as yet unexpressed WTHD in these patients.

### Implications for research

Further research on this topic could focus on new patient groups that have yet to be examined in the extant literature, for example, individuals with kidney failure, chronic respiratory disease, cardiac failure or neurodegenerative disease, as well as domiciliary and/or out-patients, the aim being to gather information that will improve the care offered to those approaching the end of life. Another potential population to study would be people living in rural areas, where resources and both cultural and contextual factors differ from those of urban areas.

Research is also needed to identify how health professionals interpret and respond to the WTHD in their patients, and the impact it may have on their attitudes and behaviour. Finally, although one of the inclusion criteria here was that the primary study had to focus on the perspective of the patient expressing the WTHD, it would be interesting to explore the meaning attributed to the WTHD by relatives and/or carers, as this complementary knowledge could help in the design of care plans.

### Conclusions

Although the development of a theoretical proposal was not the aim of this study the results of the synthesis do provide an explanatory model of the WTHD that is common to people from different countries and healthcare systems. Indeed, patients who express the WTHD show commonalities in how they experience their illness. Moreover, the synthesis has identified the elements that seem to be required in order to understand the needs of these patients, and to develop individualized care plans for them.
